# Assessment of Oxidative Stress Biomarkers in Rat Blood

**DOI:** 10.21769/BioProtoc.4626

**Published:** 2023-03-05

**Authors:** Yuri K. Sinzato, Thiago Rodrigues, Larissa L. Cruz, Vinícius S. Barco, Maysa R. Souza, Gustavo T. Volpato, Débora C. Damasceno

**Affiliations:** 1 Laboratory of Experimental Research on Gynecology and Obstetrics, Botucatu Medical School, São Paulo State University (Unesp), Botucatu, São Paulo State, Brazil; 2 Center of Natural and Human Sciences (CCNH), Federal University of ABC (UFABC), Santo André, São Paulo State, Brazil; 3 Laboratory of System Physiology and Reproductive Toxicology, Institute of Biological and Health Sciences, Federal University of Mato Grosso (UFMT), Barra do Garças, Mato Grosso State, Brazil

**Keywords:** Oxidative stress, Lipid peroxidation, Biomarker, Redox status, Pregnancy

## Abstract

Redox status assessments are time-consuming, require a large volume of samples and great reagent amounts, and are not adequately described for methodological reproducibility. Here, the objective was to standardize redox balance determination, based on previously described spectrophotometric tests in pregnant rats, to improve precision, time dispensed, and the volume of samples and reagents, while maintaining accuracy and adequate cost benefits. This protocol summarizes oxidative stress markers, which focus on spectrophotometric tests for the assessment of thiobarbituric acid–reactive substances, reduced thiol groups, and hydrogen peroxide, as well as the antioxidant activity of superoxide dismutase, glutathione peroxidase, and catalase in washed erythrocyte and serum samples from full-term pregnant rats. For non-pregnant rats and other species, it is necessary to standardize these determinations, especially the sample volume. All measurements were normalized by the estimated protein concentrations in each sample. To establish optimum conditions for the reproducibility of the proposed methods, we describe all changes made in each assay’s steps based on the reference method reassessed for the new standardizations. Furthermore, the calculations of the concentrations or activities of each marker are presented. Thus, we demonstrate that the analysis of serum samples is easier and faster, but it is impossible to detect catalase activity. Furthermore, the proposed methods can be applied for redox balance determination, especially using smaller reagent amounts and lower sample volumes in lesser time without losing accuracy, as is required in obtaining samples during rat pregnancy.

## Background

Reactive oxygen species (ROS) are free radicals; their non-radical intermediates, produced in a limited amount in response to physiological stimuli, mediate inter- and intracellular signaling (Fujii et al., 2005). Hydroxyl radicals are free radicals that are capable of causing lipid peroxidation in the plasma membrane or in organelles that contain large quantities of polyunsaturated fatty acid side chains (Burton and Jauniaux, 2011). Lipid peroxidation is mostly assessed by measuring thiobarbituric acid–reactive substance (TBARS) concentration (De Souza et al., 2010). Furthermore, amino acids are also a target for oxidative damage. The extraction of hydrogen ions from the thiol group of cysteine can form disulfide bonds and abnormal protein folding, which can lead to functional impairment and protein aggregation (Burton and Jauniaux, 2011). To assess reduced thiol concentrations, the determination of the content of reduced sulfhydryl groups (-SH) provides useful and early indications of antioxidant capacity and structural and functional alterations in the cell membrane ( Kumar and Maurya, 2013).

Another oxygen-free radical is the superoxide anion (O_2_-), which is mainly generated by mitochondria and the endoplasmic reticulum (Burton and Jauniaux, 2011). The superoxide anion is detoxified by superoxide dismutase (SOD) enzymes, which convert O_2_- to hydrogen peroxide (H_2_O_2_). Hydrogen peroxide is not a free radical, making it less reactive than the superoxide anion. However, it comes under the term ROS as it is intimately involved in the generation and detoxification of free radicals. H_2_O_2_is, in turn, detoxified to water by the enzymes catalase (CAT) and glutathione peroxidase (GSH-Px) (Burton and Jauniaux, 2011). An imbalance between ROS production and antioxidant defenses causes oxidative stress, which might damage macromolecular classes (lipids, protein, and DNA), leading to loss of function and even cell death (Burton and Jauniaux, 2011; Birben et al., 2012).

The redox status is an important measurement to evaluate pathophysiological mechanisms involved in several disorders in pregnancy (Agarwal et al., 2005 and 2012). Several methods to assess redox status in reproductive processes have been reported, but the type of sample and techniques employed for oxidative stress measurement have yielded conflicting results that hinder comparisons. Most of these studies have measured total antioxidant capacity (TAC) (Pisoschi and Negulescu, 2012). However, the measurement of single components or TAC alone may not be a reliable indicator of oxidative stress under physiological and pathological conditions. Investigating the scavenging activity of antioxidant enzymes such as SOD, GSH-Px, and CAT might be very useful, as the target oxygen radicals of each of these enzymes would be known. Techniques such as spectrophotometry, fluorimetry, and high-performance liquid chromatography have been used for assessing oxidative stress and require a large sample volume and great reagent amounts, along with being very time-consuming ( Wang and Joseph, 1999; Del Rio et al., 2005; Ćebović et al., 2013). Spectrophotometry, however, seems to be the most easily available and cost-effective technique.

Other studies must be performed to develop more accurate and low-cost techniques that are relevant for oxidative stress evaluation. Furthermore, investigators need blood samples for the determination of different biomarkers during pregnancy in rats. However, there are many methodologies that use a large volume for each marker, making it difficult to determine the different biomarkers in the same animal. Therefore, our objective was to standardize the redox balance determination based on previously described spectrophotometric tests in pregnant Wistar/Sprague-Dawley rats, to improve precision, time dispensed, and the volume of samples and reagents concentrations, while maintaining accuracy and adequate cost-benefits. Additionally, sample collection and processing steps are described in detail to provide reproducibility.

## Materials and Reagents

1. Paper towel (Garden^®^, catalog number: 6610)
2. 50 × 50 cm filter paper (Qualy^®^, catalog number: 1282)
3. Laboratory film (Parafilm^®^, catalog number: PM-996)
4. 1.5 mL microtube (Eppendorf^®^, catalog number: 0030120086)
5. 2.0 mL microtube (Eppendorf^®^, catalog number: 0030120094)
6. 0.1–10 μL tip (Eppendorf^®^, catalog number: 0030000838)
7. 2–200 μL tip (Eppendorf^®^, catalog number: 0030000889)
8. 50–1,000 μL tip (Eppendorf^®^, catalog number: 0030000927)
9. 96-well microplate (Corning^®^, catalog number: 3595)
10. Quartz cuvettes (Daigger Scientific^®^, catalog number: EF22153L/FX22153L)
11. 10 mL volumetric flask (ISO: 3819, Laborglas^®^, catalog number: 9110608)
12. 50 mL volumetric flask (ISO: 3819, Laborglas^®^, catalog number: 9110617)
13. 100 mL volumetric flask (ISO: 3819, Laborglas^®^, catalog number: 9110624)
14. 250 mL volumetric flask (ISO: 3819, Laborglas^®^, catalog number: 9110636)
15. 600 mL volumetric flask (ISO: 3819, Laborglas^®^, catalog number: 9110648)
16. 1,000 mL volumetric flask (ISO: 3819, Laborglas^®^, catalog number: 910654)
17. 10 mL beaker (ISO: 4788, Laborglas^®^, catalog number: 9139608)
18. 100 mL beaker (ISO: 4788, Laborglas^®^, catalog number: 9139624)
19. 250 mL beaker (ISO: 4788, Laborglas^®^, catalog number: 9139636)
20. 500 mL beaker (ISO: 4788, Laborglas^®^, catalog number: 9139744)
21. 1,000 mL beaker (ISO: 4788, Laborglas^®^, catalog number: 9139754)
22. Glass sticker (Laborglas^®^, catalog number: 9108805)
23. 1,000 mL Amber bottle (Laborglas^®^, catalog number: 91806545)
24. Purified water
25. 2-mercaptoethanol (purity ≥99%) (Sigma-Aldrich^®^, catalog number: 8057400250)
26. 2-thiobarbituric acid (TBA, purity ≥98%) (Sigma-Aldrich^®^, catalog number: T5500)
27. 5,5-dithiobis (2-nitro-benzoic acid) (DTNB, purity ≥ 98%) (Sigma-Aldrich^®^, catalog number: D8130)
28. 5-sulfosalicylic acid hydrate (purity ≥95%) (Sigma-Aldrich^®^, catalog number: 390275)
29. Bovine serum albumin (BSA) (Sigma-Aldrich^®^, catalog number: A-4503)
30. Calcium chloride (CaCl_2_, A.C.S.) (Sigma-Aldrich^®^, catalog number: C1016)
31. Ethylenediaminetetraacetic acid (EDTA, purity ≥99%) (Sigma-Aldrich^®^, catalog number: S26-36)
32. Glutathione reductase (GSH-Rd, 100–300 units/mg protein) (Sigma-Aldrich^®^, catalog number: G3664)
33. Horseradish peroxidase (HRP) (25,000 units) (Sigma-Aldrich^®^, catalog number: P8250)
34. L-glutathione reduced (GSH, purity 98%) (Sigma-Aldrich^®^, catalog number: G4251)
35. Magnesium chloride (MgCl_2_, A.C.S.) (Sigma-Aldrich^®^, catalog number: 208337)
36. Phenol red (Sigma-Aldrich^®^, catalog number: 114529)
37. Phosphate buffered saline (PBS, 0.138 M NaCl, 0.0027 M KCl, pH 7.4) (Sigma-Aldrich^®^, catalog number: P3813)
38. Potassium cyanide (purity ≥97%) (Sigma-Aldrich^®^, catalog number: 31252)
39. Potassium ferricyanide (purity ≥99%) (Sigma-Aldrich^®^, catalog number: 702587)
40. Sodium hydroxide (NaOH, purity ≥97%) (Sigma-Aldrich^®^, catalog number: 221465)
41. Sodium phosphate monobasic monohydrate (NaH_2_PO_4_ ·H_2_O, A.C.S.) (Sigma-Aldrich^®^, catalog number: S9638)
42. *
T* -butyl hydroperoxide 70% aqueous solution (Sigma-Aldrich^®^, catalog number: B2633)
43. β-nicotinamide adenine dinucleotide 2′-phosphate reduced tetrasodium salt hydrate (NADPH, purity ≥93%) (Sigma-Aldrich^®^, catalog number: N1630)
44. Protein assay dye reagent concentrate (Bio-Rad^®^, catalog number: 500-0006)
45. TRIS-Ultrapure ^TM^ (purity ≥99.9%) (Invitrogen^®^, catalog number: 15504-020)
46. Hydrochloric acid (HCl, A.C.S., purity 36.5%–38%) (Synth^®^, catalog number: A1028.01.BJ)
47. Pyrogallic acid (purity ≥99.9%) (Synth^®^, catalog number: A1052.01.AE)
48. Di-potassium hydrogen phosphate trihydrate (K_2_HPO_4_ , purity ≥99%) (Merck^®^, catalog number: AO150099.010)
49. Ethyl alcohol (A.C.S., purity 99.5%) (Merck^®^, catalog number: 108543)
50. Sodium bicarbonate (NaHCO _3_ , purity ≥99.5%) (Merck^®^, catalog number: 6329)
51. Potassium chloride (KCl, A.C.S.) (Vetec^®^, catalog number: 104)
52. Sodium chloride (NaCl, A.C.S.) (Vetec^®^, catalog number: V003132)
53. Hydrogen peroxide (H_2_O_2_, A.C.S., purity 35%) (Dinâmica^®^, catalog number: 1857)
54. Potassium dihydrogen phosphate (KH_2_PO_4_ , A.C.S.) (Dinâmica^®^, catalog number: P.10.0513.009.00)
55. Sodium phosphate dibasic (Na_2_HPO_4_ , A.C.S.) (Dinâmica^®^, catalog number: P.10.0513.012.00)
56. Stabilizing solution (2.7 mM EDTA and 0.7 mM 2-mercaptoethanol) (see Recipes)
57. Drabkin’s solution (see Recipes)
58. 1 μg/μL stock solution (see Recipes)
59. 3% (w/v) 5-sulfosalicylic acid hydrate (for 100 samples) (see Recipes)
60. Thiobarbituric acid solution (TBA) 0.67% (for 200 samples) (see Recipes)
61. 1 M HCl (see Recipes)
62. 0.1 M TRIS/HCl 0.5 mM EDTA pH 8.0 (for 250 duplicate sample analysis) (see Recipes)
63. 10 mM DTNB (for 250 duplicate washed erythrocytes samples and 100 duplicate serum samples) (see Recipes)
64. Solution 1: 1.71 M NaCl, 34 mM KCl, 14 mM K_2_HPO_4_ , 0.1 M Na_2_HPO_4_ (see Recipes)
65. Solution 2: 90 mM CaCl_2_(see Recipes)
66. Solution 3: 0.11 M MgCl_2_(see Recipes)
67. Solution 4: 0.1% phenol red (see Recipes)
68. Phosphate buffer solution A (see Recipes)
69. Peroxidase solution (HRP) (see Recipes)
70. Buffer solution b (see Recipes)
71. 1 M NaOH (see Recipes)
72. 1 M TRIS/HCl 5 mM EDTA pH 8.0 (for 150 duplicate sample analysis) (see Recipes)
73. 10 mM pyrogallol solution (for 250 duplicate sample analysis) (see Recipes)
74. 50 mM TRIS/HCl 5 mM EDTA (for 500 duplicate sample analysis) (see Recipes)
75. 1% NaHCO _3_ and 1 mM EDTA (see Recipes)
76. 10 mM HCl solution (see Recipes)
77. 0.1 M glutathione reduced (GSH) (for 60 duplicate sample analysis) (see Recipes)
78. 10 U/mL glutathione reductase (GSH-Rd) (for 60 duplicate sample analysis) (see Recipes)
79. 7 mM *t* -butyl hydroxiperoxide (for 125 duplicate sample analysis) (see Recipes)
80. 4 mM NADPH (for 60 duplicate sample analysis) (see Recipes)
81. 0.1 M NaH_2_PO_4_ ·H_2_O (for 200 samples analysis) (see Recipes)
82. 1 M hydrogen peroxide (H_2_O_2_) (see Recipes)

## Equipment

1. 0.1–2.5 μL micropipette (Eppendorf^®^, catalog number: 4924000010)
2. 0.5–10 μL micropipette (Eppendorf^®^, catalog number: 4924000029)
3. 2–20 μL micropipette (Eppendorf^®^, catalog number: 4924000045)
4. 10–100 μL micropipette (Eppendorf^®^, catalog number: 4924000053)
5. 20–200 μL micropipette (Eppendorf^®^, catalog number: 4924000061)
6. 100–1,000 μL micropipette (Eppendorf^®^, catalog number: 4924000088)
7. Centrifuge (Eppendorf^®^, model: 5804R)
8. Water purifier (GEHAKA^®^, Master System)
9. Microplate reader (Biotek^®^, Power Wave XS)
10. Spectrophotometer (Shimadzu^®^, model: UV1800)
11. Digital microplate mixer (IKA^®^, model: MS3)
12. Water bath (Quimis^®^, model: Q334M-24)
13. Analytical balance (Denver Instrument^®^, model: APX200)
-80 °C ultra-freezer (ColdLab^®^, model: CL200-86V)
15. Refrigerator (Electrolux^®^, model: RDE 32)
16. Vortex agitator mixer (Quimis^®^, model: G220)
17. Magnetic agitator (Quimis^®^, model: Q2461-22)
18. pH meter (Tecnal^®^, model: Tec05)
19. Autoclave (Ecel^®^, model: EC 21D)
20. Fume hood (Permution^®^, model: NBR 7094)

## Software

1. Gen5 (Bio Tek^®^)
2. UVProbe (Shimadzu^®^)
3. Statistica (StatSoft^®^)
4. Microsoft Office 365 (Microsoft^®^)

## Procedure

- **
Sample collection
**
  1. Collect whole blood samples from pregnant rats at term through a cut at the end of the tail in both dry/free of anticoagulant (approximately 5.0–9.0 mL of blood) and heparinized (approximately 1.5 mL of blood in 0.2 mL of heparin) tubes. These volumes of blood are sufficient to process samples for oxidant and antioxidant biomarker measurements.
  2. Store under refrigeration at 4–8 °C for 30 min after collection.
*
Note: The samples cannot be stored on wet ice.
*
  3. Use the heparinized tubes to obtain washed erythrocyte samples (Figure 1):
    **Figure 1.**
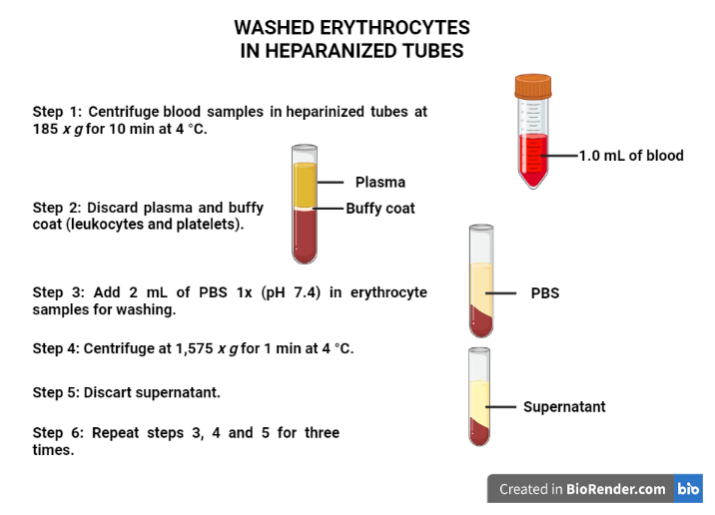
Procedures to obtain the washed erythrocytes. Created with BioRender.com.
    - Centrifuge the blood samples in heparinized tubes at 185*× g*for 10 min at 4 °C.
    - Discard the plasma (light yellow supernatant) and the buffy coat (leukocytes and platelets, translucid supernatant).
*Notes:*
*1) If the phases do not separate appropriately, repeat centrifuging. If the phases do not separate again, discard the sample.*
*2) If there is high lipid concentration in the samples, the plasma color will be white.*
    - Add 2 mL of PBS 1× (pH 7.4) to the erythrocyte samples for washing.
    - Centrifuge at 1,575*× g*for 1 min at 4 °C.
    - Discard the supernatant (translucid supernatant).
    - Repeat procedures A3c–e three times.
    - Identify three microtubes for Hb, TBARS, and SH level measurements (Figure 2):
      i) Pipette 50 μL of washed erythrocyte samples (pellet obtained from previous steps) directly in each microtube (three in total) for later measurements of Hb, TBARS, and SH levels.
      ii) Add 950 μL of purified water in each microtube containing 50 μL of the washed erythrocyte.
      iii) Gently homogenize the samples by inversion.
      iv) Store aliquots at -80 °C until assays (stable for up to six months).
      **Figure 2.**
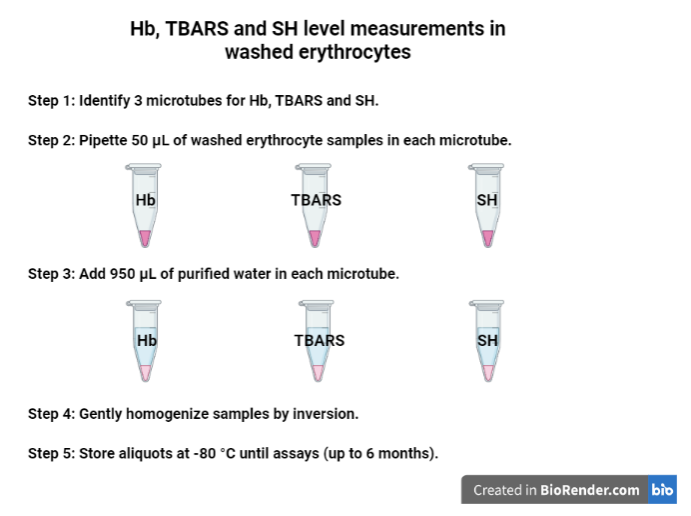
Aliquots for Hb, TBARS, and SH level measurements in the washed erythrocytes using purified water. Created with BioRender.com.
    - Identify five microtubes for Hb, SOD, H_2_O_2_, GSH-Px, and CAT level measurements (Figure 3):
      i) Pipette 50 μL of the washed erythrocyte samples (pellet obtained from previous steps) directly in each microtube (five in total) for activity measurements of Hb, SOD, H_2_O_2_GSH-Px, and CAT.
      ii) Add 950 μL of the stabilizing solution (see Recipe 1) in each microtube.
      iii) Gently homogenize the samples by inversion.
      iv) Store the aliquots at -80 °C until assays (stable for up to six months).
    **Figure 3.**
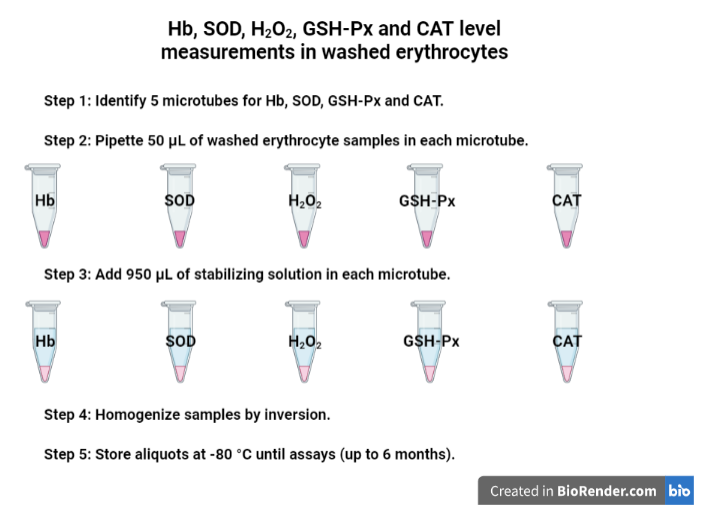
Aliquots for Hb, SOD, H_2_O_2_, GSH-Px, and CAT level measurements in the washed erythrocytes using a stabilizing solution. Created with BioRender.com.
  4. Serum sample collection in dry/free anticoagulant tubes (mentioned in step 1,Figure 4):
    - Centrifuge the blood samples in dry tubes at 1,575*× g*for 10 min at 4 °C.
    - Collet the serum using a micropipette and put it in another tube. Next, discard the pellet.
    - Pipette the serum aliquots directly into six microtubes for protein quantification (20 μL), TBARS (550 μL), SH (10 μL), H_2_O_2_(20 μL), SOD (5 μL), and GSH-Px (5 μL) using specific identification in each microtube.
    - Store at -80 °C until analysis (stable for up to six months).
    **Figure 4.**
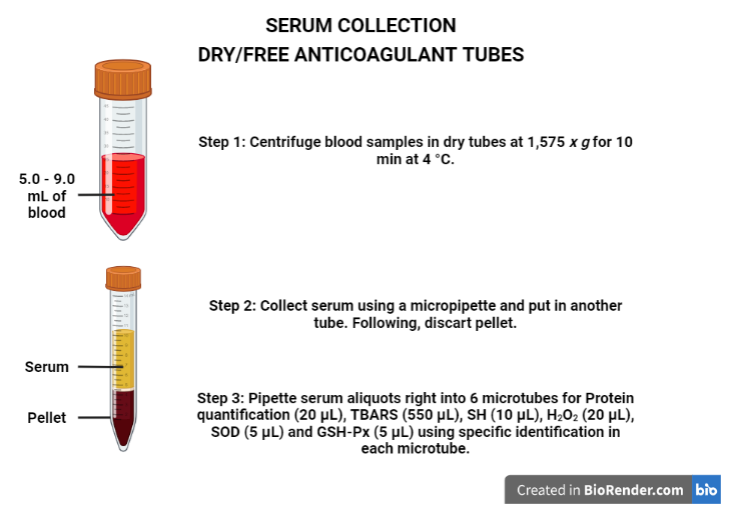
Procedures to obtain the serum. Created with BioRender.com.
- **
Hemoglobin (Hb) quantification in the washed erythrocytes
**
  1. Take identified microtubes with Hb [diluted with purified water (step A3g) and stabilizing solution (step A3h)].
*
Note: Hemoglobin concentration measurements are similar in purified water and/or stabilizing solution because they are necessary for the determination of the normalization of oxidant and antioxidant concentrations. Thus, two microplates will be used for Hb determination (one for the purified water and another for the stabilizing solution).
*
  2. Homogenize the samples in the vortex agitator mixer.
  3. Pipette 200 μL of Drabkin’s solution (see Recipe 2) in two wells of the microplate as a blank.
  4. Pipette 197.5 μL of Drabkin’s solution and 2.5 μL of samples (washed erythrocytes) in each well of the microplate (in duplicates).
  5. Cover the microplate and mix using a microplate mixer for 1 min at 750 rpm for homogenization.
  6. Rest the microplate for 10 min at 25 °C.
  7. Lightly tap the sides of the microplate several times to gently mix.
  8. Measure the absorbance at a wavelength of 546 nm using Gen5 software.
  9. Calculate Hb concentrations (see Data analysis).
- **
Quantification of protein concentrations in serum samples by Bradford’s method (Bradford, 1976)
**
  1. Dilute one part of the serum samples in 100 parts of purified water in a microtube.
*
Note: Other dilutions can be tested according to the experiment.
*
  2. Identify six microtubes for the BSA solutions: 0, 0.1, 0.2, 0.4, 0.8, and 1.0 μg/μL.
  3. Prepare the BSA solutions for a standard curve setting from the 1 μg/μL stock solution (see Recipe 3), pipetting the following volumes of BSA and purified water:
    **Table:** 

Concentration (µg/µL)	Stock solution (µL)	Purified water (µL)
0	0	50
0.1	5	45
0.2	10	40
0.4	20	30
0.8	40	10
1.0	50	0
  4. Dilute the protein assay dye reagent concentrate in a ratio of one part of the reagent to four parts of purified water to prepare Bradford’s reagent.
For example: dilute 10 mL of the protein assay dye reagent concentrate in 40 mL of purified water.
*
Note: Calculate the quantity for Bradford’s reagent according to the number of wells that will be used.
*
  5. Pipette 190 μL of Bradford’s reagent into each well of the microplate (standard curve and sample wells).
  6. Pipette 10 μL of each BSA solution into the standard curve wells (in duplicates). Homogenize with the tip.
  7. Pipette 10 μL of the serum samples into other wells (in duplicates). Homogenize with the tip.
  8. Measure the absorbance at a wavelength of 595 nm using Gen5 software.
  9. Calculate total protein concentrations (see Data analysis).
- **
Thiobarbituric acid reactive substances (TBARS)
**
  1. Prepare 3% (w/v) 5-sulfosalicylic acid hydrate (see Recipe 4) and TBA 0.67% (see Recipe 5) solutions.
  2. Add 500 μL of serum or washed erythrocytes to a test microtube containing 500 μL of 3% 5-sulfosalicylic acid hydrate.
  3. Vortex the microtube for 10 s.
  4. Centrifuge the microtube at 18,000 *× g* for 3 min at 4 °C.
  5. Rest the microtube for 15 min at 25 °C.
  6. Pipette the supernatant and store it in new microtubes.
*
Note: Avoid pipetting the brown granules.
*
  7. Add 500 μL of purified water in a tube as a blank.
  8. Add 500 μL of the supernatant to 500 μL of the TBA solution in a tube for each sample.
  9. Vortex the tubes for 10 s.
  10. Heat the mixtures at 95 °C for 30 min in a water bath with the tubes partially sealed.
  11. Cool on ice for 10 min to stop further reactions.
  12. Do not exceed 30 min for the next steps (a–e):
    - Equilibrate the blanks and samples at 25 °C.
    - Pipette 300 μL of purified water in two wells as a blank.
    - Pipette 300 μL of the samples into each well of the microplate (in duplicates).
    - Lightly tap the sides of the microplate several times to gently mix.
    - Measure the absorbance at a wavelength of 535 nm using Gen5 software.
  13. Calculate TBARS concentrations (see Data analysis).
- **
Reduced thiol groups (-SH)
**
  1. Prepare 0.1 M TRIS/HCl 0.5 mM EDTA pH 8.0 solution (see Recipe 7).
  2. Prepare the 10 mM DTNB solution (see Recipe 8).
  3. For the -SH quantification in the washed erythrocytes:
    - Pipette 200 μL of 0.1 M TRIS/HCl 0.5 mM EDTA pH 8.0 in two wells as a blank.
    - Pipette 195 μL of 0.1 M TRIS/HCl 0.5 mM EDTA pH 8.0 solution into other wells (in duplicates).
    - Pipette 1.0 μL of the washed erythrocyte (in duplicates except in blank wells).
    - Pipette 4.0 μL of the 10 mM DTNB solution into the sample wells (in duplicates).
    - Cover the microplate and mix using a microplate mixer for 10 min at 750 rpm for homogenization.
    - Measure the absorbance at a wavelength of 412 nm.
  4. For the -SH serum quantification:
    - Pipette 200 μL of 0.1 M TRIS/HCl 0.5 mM EDTA pH 8.0 in two wells as a blank.
    - Pipette 175 μL of 0.1 M TRIS/HCl 0.5 mM EDTA pH 8.0 solution into other wells (in duplicates).
    - Pipette 5.0 μL of serum (in duplicates except in blank wells).
    - Pipette 20 μL of the 10 mM DTNB solution into the sample wells (in duplicates).
    - Mix the microplate using a microplate mixer at 750 rpm for 10 min.
    - Measure the absorbance at a wavelength of 412 nm using Gen5 software.
  5. Calculate -SH concentrations (see Data analysis).
- **
Hydrogen peroxide (H_2_O_2_)
**
  1. Prepare the solutions below:
    - Solution 1 (see Recipe 9).
    - Solution 2 (see Recipe 10).
    - Solution 3 (see Recipe 11).
    - Solution 4 (see Recipe 12).
    - Phosphate buffer solution A (see Recipe 13).
    - Peroxidase solution (HRP) (see Recipe 14).
  2. Prepare buffer solution B (see Recipe 15).
  3. Pipette 110 μL of the buffer solution B into two wells of the microplate as a blank.
  4. For the H_2_O_2_quantification in the washed erythrocytes:
    - Pipette 5 μL of the washed erythrocyte sample into other wells (in duplicates).
    - Pipette 105 μL of the buffer solution B into the sample wells.
    - Mix the microplate using a microplate mixer at 750 rpm for 10 s.
    - Incubate the microplate at 37 °C for 10 min.
    - Pipette 10 μL of 1 M NaOH (see Recipe 16) to stop the reaction.
    - Measure the absorbance at a wavelength of 620 nm.
  5. For the H_2_O_2_quantification in the serum samples:
    - Pipette 10 μL of the serum sample into other wells (in duplicates).
    - Pipette 100 μL of the buffer solution B into the sample wells.
    - Mix the microplate using a microplate mixer at 750 rpm for 10 s.
    - Incubate the microplate at 37 °C for 10 min.
    - Pipette 10 μL of 1 M NaOH to stop the reaction.
    - Measure the absorbance at a wavelength of 620 nm using Gen5 software.
  6. Calculate H_2_O_2_concentrations (see Data analysis).
- **
Superoxide dismutase (SOD)
**
  1. Prepare 1 M TRIS/HCl 5 mM EDTA pH 8.0 solution (see Recipe 17).
  2. Prepare 10 mM pyrogallol solution (see Recipe 18).
  3. Pipette 147 μL of 1 M TRIS/HCl 5 mM EDTA pH 8.0 solution into two wells of the microplate as a blank.
  4. Pipette 144 μL of 1 M TRIS/HCl 5 mM EDTA pH 8.0 into other wells (in duplicates).
  5. Pipette 3.0 μL of the washed erythrocyte/serum (in duplicates except in the blank wells).
  6. Lightly tap the sides of the microplate several times to gently mix.
  7. Add 3.0 μL of 10 mM pyrogallol solution into all wells including the blank (30 s for pipetting the solution in all microplates).
  8. Lightly tap the sides of the microplate several times to gently mix.
  9. Measure the absorbance during the time intervals of 0 (T0) and 10 (T10) min at 25 °C at a wavelength of 420 nm.
  10. Calculate SOD concentrations using Gen5 software (see Data analysis).
- **
Glutathione peroxidase (GSH-Px or GPx)
**
  1. Prepare the solutions below:
*
Note: All solutions (except 50 mM TRIS/HCl 5 mM EDTA pH 7.6 and 7 mM* t-* butyl hydroperoxide) and reagents must be kept on ice until the moment of the assay.*
    - 0.1 M TRIS/HCl 0.5 mM EDTA pH 8.0 solution (see Recipe 7).
    - 50 mM TRIS/HCl 5 mM EDTA pH 7.6 solution (see Recipe 19).
    - 1% (w/v) NaHCO_3_and 1 mM EDTA solution (see Recipe 20).
    - 10 mM HCl solution (see Recipe 21).
    - 0.1 M glutathione reduced (GSH) solution (see Recipe 22).
    - 10 U/mL glutathione reductase (GSH-Rd) solution (see Recipe 23).
    - 7 mM*t*-butyl hydroperoxide solution (see Recipe 24).
    - 4 mM NADPH solution (see Recipe 25).
  2. Prepare a MIX solution containing:
*
Note: The MIX solution must be prepared immediately before the assay. It is recommended to trigger the reaction in up to 16 duplicate samples. If you prepare more than 16 duplicate samples, the results will be different because of the long time between the reactions.
*
    - 240 μL of the 0.1 M GSH solution.
    - 1,200 μL of the 10 U/mL GSH-Rd solution.
    - 1,200 μL of the 4 mM NADPH.
    - Keep this MIX solution on ice.
  3. Pipette 100 μL of the 50 mM TRIS/HCl 5 mM EDTA pH 7.6 solution in all wells of the microplate.
  4. Pipette 1.0 μL of the serum or 0.5 μL of the washed erythrocytes into all wells (in duplicates).
  5. Lightly tap the sides of the microplate several times to gently mix.
  6. Pipette 50 μL of the MIX solution into all wells.
  7. Lightly tap the sides of the microplate several times to gently mix.
  8. Pipette 20 μL of the 7 mM t-butyl hydroperoxide solution into all wells (to initiate a reaction).
*
Note: It takes 15 s to pipette the 7 mM t-butyl hydroperoxide solution into the sample wells and trigger the reaction in up to 16 duplicate samples.
*
  9. Quickly homogenize the microplate.
  10. Measure the absorbance every one-minute interval for 5 min at 25 °C in a microplate at a wavelength of 340 nm using Gen5 software.
  11. Calculate GSH-Px concentrations (see Data analysis).
- **
Catalase (CAT)
**
  1. Prepare 0.1 M NaH_2_PO_4_ ·H_2_O solution (see Recipe 26).
  2. Prepare 1 M H_2_O_2_solution (see Recipe 27).
  3. Verify hydrogen peroxide molarity:
    - Pipette 1,000 μL of the 0.1 M NaH_2_PO_4_ ·H_2_O solution into a quartz cuvette (cuvette 1) as a blank in the reference position.
    - Pipette 990 μL of the 0.1 M NaH_2_PO_4_ ·H_2_O solution into another quartz cuvette (cuvette 2) in the reading position.
    - Select AUTO ZERO in a UV1800 spectrophotometer for calibration.
    - Wait until the absorbance reaches zero.
    - Keep the cuvette 1 in the reference position.
    - Add 10 μL of 1 M H_2_O_2_into cuvette 2.
    - Quickly homogenize.
    - Return cuvette 2 to the reading position.
    - Measure the absorbance at a wavelength of 240 nm.
    - Calculate the hydrogen peroxide real molarity (see Data Analysis and Notes).
  4. Wash both cuvettes.
  5. Pipette 999 μL of the 0.1M NaH_2_PO_4_ ·H_2_O solution with 1.0 μL of washed erythrocytes into a quartz cuvette (cuvette 1) as a blank (in the reference position).
  6. Pipette 978 μL of the 0.1M NaH_2_PO_4_ ·H_2_O solution with 1.0 μL of the washed erythrocytes into another quartz cuvette (cuvette 2) in the reading position.
  7. Select AUTO ZERO in a UV1800 spectrophotometer.
  8. Wait until the absorbance reaches zero.
  9. Add 21 μL of 1 M H_2_O_2_into the quartz cuvette 2 for a quick homogenization.
  10. Perform the kinetics measurements for 2 min at 25 °C in a UV1800 spectrophotometer at 240 nm using UVProbe software.
  11. Calculate CAT concentrations (see Data analysis).

## Data analysis

To ensure sample homogeneity, all measurements were adjusted according to the protein concentration estimated in each sample (hemoglobin and total protein for erythrocytes and serum, respectively).

**Assays**

To determine the minimum sample volume and reagent concentrations required to reproduce each reference method in pregnant rats at term, decreasing amounts of reagents were tested until the results obtained were equal to the reference values of other articles established in the literature. All samples were analyzed in duplicates at 25 °C.

**Hemoglobin**

Absorbance, expressed in grams (g) of hemoglobin per deciliter (dL), was measured at 546 nm ( Tentori and Salvati, 1981) and calculated using the extinction coefficient of the complex cyanmethemoglobin (hemoglobin-cyanide) monomer at 546 nm (11.0 mM ^-1^ cm ^-1 ^) ( Assendelft and Zijlstra, 1975) and the pathlength (0.6 cm) of the solution in the well, using the Gen5 software.


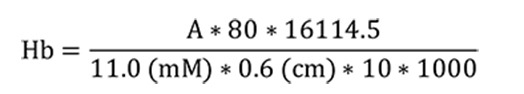


**Hb =** hemoglobin concentration in g/dL

**A =** mean sample absorbance – blank absorbance

**80 =** dilution factor (Drabkin’s solution volume/sample volume)

**16114.5 =** hemoglobin monomer molar mass (complex cyanmethemoglobin–hemoglobin–cyanide monomer at 546 nm)

**10 =** conversion from L to dL

**1000 =** conversion from mg to g

**Total protein concentration**

Total protein absorbance values were determined by Bradford’s method (1976) for the normalization of oxidative stress biomarkers using Gen5 software. The total protein concentration is determined by the linear equation obtained from the standard curve. Standard curve is an increasing or decreasing succession of points obtained from the relationship between the concentration of the standard species and its signal intensity from the detection system. The most important part of the graph is the quadratic equation, from which the concentration can be calculated. The graph also provides the value of R ^2^ , which is the value of the angular coefficient. The closer to 1 the value of R is, the better is the line described by the linear regression of the points aiming to obtain lines ≥ 0.99. Quantification requires knowing the dependence between the measured response and the concentration of the analyte. Linearity is obtained by internal or external standardization and formulated as a mathematical expression used to calculate the concentration of the analyte to be determined in the real sample. The equation of the line that relates the two variables is:

y=ax+b

**y** = measured response (absorbance)

**x** = concentration

**a** = slope of the analytical curve = sensitivity

**b** = intersection with the y axis when x = 0

**Figure 5.**
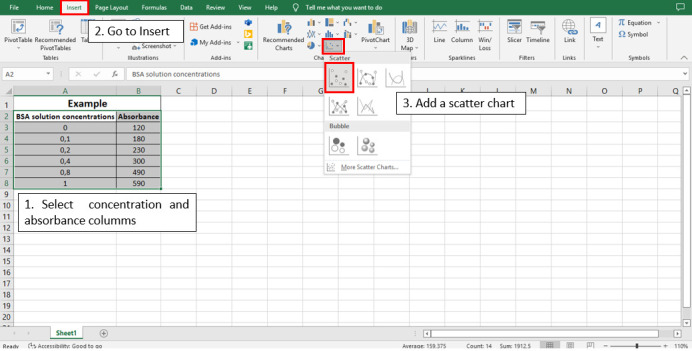
Steps 1, 2, and 3 to calculate the line equation from the standard curve

**Figure 6.**
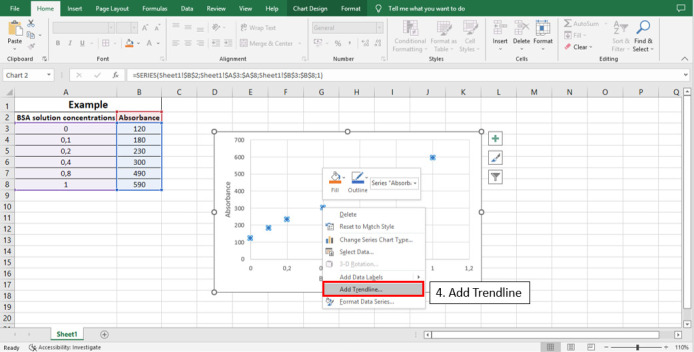
Step 4 to calculate the line equation from the standard curve

**Figure 7.**
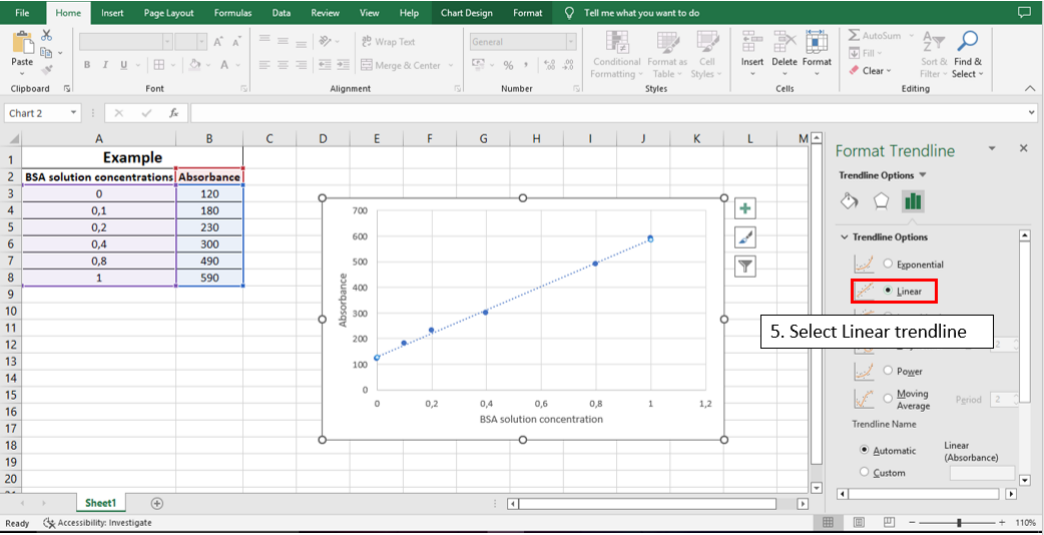
Step 5 to calculate the line equation from the standard curve

**Figure 8.**
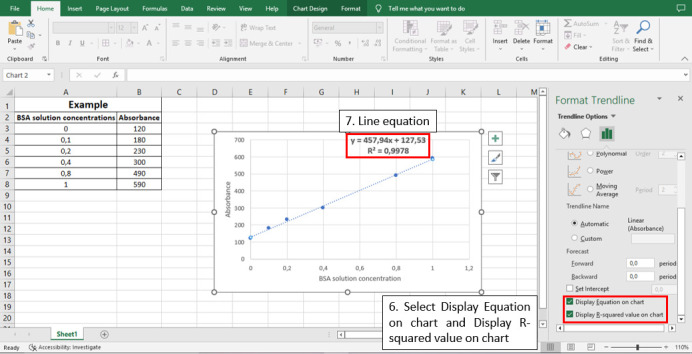
Steps 6 and 7 to calculate the line equation from the standard curve

To calculate the linear equation from the standard curve in Microsoft Office Excel^®^, follow the steps depicted in Figures 5 –8:

**y** = measured response (absorbance)

**x** = total protein concentration in μg/μL

**457.94** = slope of the analytical curve = sensitivity

**127.53** = intersection with the y axis when x = 0

**0.9978** = angular coefficient

*Note: If R ^2^ value < 0.98, repeat the standard curve measurements.*

To calculate total protein concentration in serum samples, follow the example below:

Considering an absorbance of 500 of the serum sample, the calculation is:

y=457.94x+127.53

500=457.94x+127.53

x=(500-127.53)/457.94

**x** = 0.81 μg/μL of total protein in the sample. To convert μg/μL to g/dL, divide the concentration value by 10.

*Note: If the absorbance value of the serum sample is higher than the absorbance of the 1.0 BSA solution concentration (standard curve), a dilution of 1:2 with purified water must be performed in the serum sample and the absorbance must be measured again. Then, the total protein concentration value (g/dL) must be multiplied by the dilution factor (in this case, multiplied by 2).*

**Thiobarbituric acid reactive substances (TBARS)**

Absorbance was measured at 535 nm using Gen5 software. Results were expressed as nM of TBARS per milligram of Hb or total protein (nM/mg Hb or total protein) in washed erythrocyte or serum samples, respectively. The MDA-TBA complex extinction coefficient of 1.56 × 105 M ^-1^ cm ^-1^ at 25 ºC ( Buege and Aust, 1978) and 0.9 cm pathlength were used for the calculations.


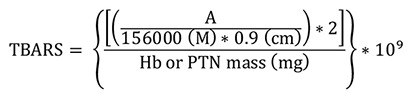


**TBARS =** Thiobarbituric acid reactive substance concentration in nM/mg Hb or total protein

**A =** mean sample absorbance – blank absorbance

**2 =** sample dilution factor

**10 ^9^ =** conversion from M to nM

**Hb =** concentration obtained by Drabkin’s solution expressed as g/dL

**Total protein (PTN) =** as PTN concentration obtained by Bradford’s method is expressed in μg/μL, it is necessary to divide this by 10 to convert to g/dL

Follow the steps below to obtain Hb or PTN mass (mg):

To convert g/dL (Hb or PTN concentrations) to mg/mL, it is necessary to multiply by 10 (g to mg and dL to mL). In addition, the used volume (0.5 mL) must be inserted by multiplication after the last conversion.

Finally, the total conversion is: Hb or PTN concentration (g/dL x 10) = Hb or PTN concentration (mg/mL x 0.5 mL) = Hb or PTN mass (mg)

**Reduced thiol groups (-SH)**

Absorbance was measured at 412 nm using Gen5 software. The results were expressed as mM of SH per mg Hb or total protein (mM/mg of Hb or total protein). The 5-thio-2-nitrobenzoic acid (TNB) extinction coefficient (14150 M ^-1^ /cm ^-1^ at 25°C) ( Riddles et al., 1983) and a 0.6 cm pathlength were used in the calculations.


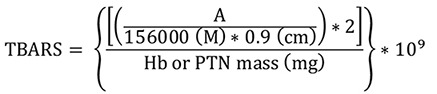


**SH =** concentration in mM/mg Hb or total protein

**A =** mean sample absorbance – blank absorbance

**10 ^3^ =** conversion from M to mM

**Hb =** concentration obtained by Drabkin’s solution expressed as g/dL

**PTN =** as PTN concentration obtained by Bradford’s method is expressed as μg/μL, it is necessary to divide this by 10 to convert to g/dL

Follow the steps below to obtain Hb or PTN mass (mg):

To convert g/dL (Hb or PTN concentrations) to mg/mL, it is necessary to multiply by 10 (g to mg and dL to mL). In addition, the sample volume used in the assay (mL) must be inserted by multiplication after the last conversion

Finally, the total conversion is: Hb or PTN concentration (g/dL x 10) = Hb or PTN concentration (mg/mL x sample volume used in the assay) = Hb or PTN mass (mg)

**Hydrogen peroxide (H_2_O_2_)**

Absorbance was measured at a wavelength of 620 nm using Gen5 software. The results were expressed as µM of H_2_O_2_per mg of hemoglobin or total protein (µM/mg Hb or total protein). The extinction coefficient of phenol red oxidation by H_2_O_2_was calculated from an adjusted standard curve in our laboratory at 37 °C (0.0296 µM ^-1^ cm ^-1 ^) and 0.33 cm pathlength.


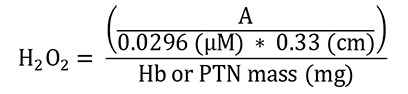


**H_2_O_2_=** concentration in µM/mg Hb or total protein

**A =** mean sample absorbance – blank absorbance

**Hb =** concentration obtained by Drabkin’s solution expressed as g/dL

**PTN =** as PTN concentration obtained by Bradford’s method is expressed as μg/μL, it is necessary to divide this by 10 to convert to g/dL

Follow the steps below to obtain Hb or PTN mass (mg):

To convert g/dL (Hb or PTN concentrations) to mg/mL, it is necessary to multiply by 10 (g to mg and dL to mL). In addition, the sample volume used in the assay (mL) must be inserted by multiplication after the last conversion.

Finally, the total conversion is: Hb or PTN concentration (g/dL x 10) = Hb or PTN concentration (mg/mL x sample volume used in the assay) = Hb or PTN mass (mg)

**Superoxide dismutase (SOD)**

Absorbance was immediately measured during the time intervals of 0 (T0) and 10 (T10) min at 25 °C in a microplate at 420 nm using Gen5 software. For the calculations, the absorbances of the blanks and samples at times T0 and T10 were used. The activity of SOD is calculated indirectly by the percentage of inhibition of pyrogallol by the sample. Therefore, the calculations were made following these steps:

1. Calculation of the percentage of inhibition of the pyrogallol autoxidation by the SOD samples:

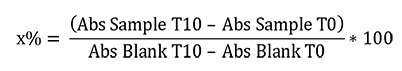

**
x% =** percentage of pyrogallol autoxidation by SOD sample
Thus, the inhibition of pyrogallol autoxidation is equal to 100 – x%.
2. Calculation of SOD activity considering that 1 IU is the volume capable of inhibiting 50% of the pyrogallol oxidation ( Marklund and Marklund, 1974):

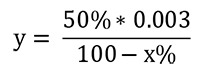

**
y =** sample volume (mL) that inhibits 50% of pyrogallol autoxidation
**
50% =** percentage inhibition of pyrogallol that equals 1 U of SOD
**
0.003 =** volume (mL) of the sample in the assay
**
100-x% =** percentage of pyrogallol inhibition by the sample
3. SOD enzymatic activity:

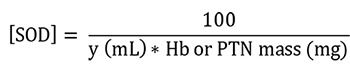

**
[SOD] =** enzymatic activity in IU/mg Hb
**
Y =** sample volume (mL) that inhibits 50% of pyrogallol autoxidation
**
100 =** conversion factor from dL to mL referring to the value of Hb (1 dL = 100 mL)
**
Hb** = concentration obtained by Drabkin’s solution expressed as g/dL
**
PTN** = as PTN concentration obtained by Bradford’s method is expressed as μg/μL, it is necessary to divide this by 10 to convert to g/dL
Follow the steps below to obtain Hb or PTN mass (mg):
To convert g/dL (Hb or PTN concentrations) to mg/mL, it is necessary to multiply by 10 (g to mg and dL to mL). In addition, the sample volume used in the assay (mL) must be inserted by multiplication after the last conversion.
Finally, the total conversion is: Hb or PTN concentration (g/dL x 10) = Hb or PTN concentration (mg/mL x sample volume used in the assay) = Hb or PTN mass (mg)
**
Glutathione peroxidase (GSH-Px)
**
Absorbance change (ΔA _340 _) per min was calculated using Gen5 software and the results were expressed as mM of GSH-Px/min/mg Hb or total protein. The NADPH extinction coefficient (6.220 M ^-1^ cm ^-1^ at 25 °C) ( Fruscione et al., 2008) and 0.5 cm pathlength were used for calculations.

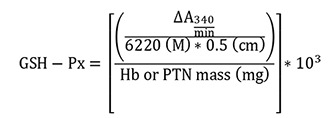

**
GSH-Px =** activity in mM/min/mg Hb or total protein
**
ΔA _340/min_ =** mean delta absorbance
**
10 ^3^ =** conversion from M to mM
**
Hb =** concentration obtained by Drabkin’s solution expressed as g/dL
**
PTN =** as PTN concentration obtained by Bradford’s method is expressed as μg/μL, it is necessary to divide this by 10 to convert to g/dL
Follow the steps below to obtain Hb or PTN mass (mg):
To convert g/dL (Hb or PTN concentrations) to mg/mL, it is necessary to multiply by 10 (g to mg and dL to mL). In addition, the sample volume used in the assay (mL) must be inserted by multiplication after the last conversion.
Finally, the total conversion is: Hb or PTN concentration (g/dL x 10) = Hb or PTN concentration (mg/mL x sample volume used in the assay) = Hb or PTN mass (mg)
**
Catalase (CAT)
**
**
Hydrogen peroxide molarity:
**
After verifying the H_2_O_2_absorbance, perform the equation to know the solution molarity. For example, the H_2_O_2_absorbance was 0.416 and the molar absorptivity of H_2_O_2_at 25 °C is 43.6. Therefore:
C=A/ε → C=0.416/43.6 → C = 0.0095 M = 9.5 mM
**
C =** concentration (mMol/L)
**
A =** Absorbance
**
Ε =** Molar absorptivity (M ^-1 ^)
Thus, although the solution concentration in the cuvette should be 10 mM (theoretical value), it is instead 9.5 mM.
To correct the solution concentration, perform the following equation:
**
C _1_ × V _1_ = C_2_× V_2_**
**
C _1_ =** Real concentration
**
V _1_ =** Volume to be pipetted
**
C_2_=** Concentration of the prepared H_2_O_2_solution
**
V_2_=** Volume to be pipetted in the cuvette 2
9.5 mM × **V _1_** = 10 mM × 10 µL
**
V _1_** = 10.5 µL – volume that must be pipetted in the cuvette to obtain a final solution at 10 mM.
In the assay, the final concentration of H_2_O_2_must be 20 mM. Therefore, the volume of the solution that will be pipetted in the cuvette will be 21 µL ( **V** _1_ × 2).
**
CAT activity
**
Absorbance change per min was calculated using UVProbe software from UV1800 spectrophotometer and the results expressed as mM of CAT/min/mg Hb (mM/min/mg Hb). The H_2_O_2_extinction coefficient (43.6 M ^-1^ cm ^-1^ at 25 °C) ( Noble and Gibson, 1970) and a 1.0 cm pathlength were used for the calculations.

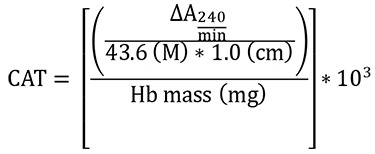

**
CAT =** activity in mM/min/mg of Hb = ∆A _240/min_ mean delta absorbance
**
10 ^3^ =** conversion from mol to mmol
**
Hb =** concentration obtained by Drabkin’s solution expressed as g/dL
Follow the steps below to obtain Hb mass (mg):
To convert g/dL (Hb concentration) to mg/mL, it is necessary to multiply by 10 (g to mg and dL to mL). In addition, the sample volume used in the assay (mL) must be inserted by multiplication after the last conversion.
Finally, the total conversion is: Hb concentration (g/dL x 10) = Hb concentration (mg/mL x sample volume used in the assay) = Hb mass (mg)
All data was obtained and analyzed using the software Statistica (StatSoft). A p-value ≤ 0.05 was considered statistically significant.
**
Results
**
The mean total protein and hemoglobin concentrations are presented in Figure 9 . These measurements were used to normalize the activity of antioxidant enzymes and redox status markers concentrations in serum and washed erythrocytes, respectively. Hemoglobin concentrations in samples diluted into a stabilizing solution were higher compared to hemoglobin concentrations diluted into purified water (p = 0.002, *t* -test) ( Figure 9).
  **Figure 9.**
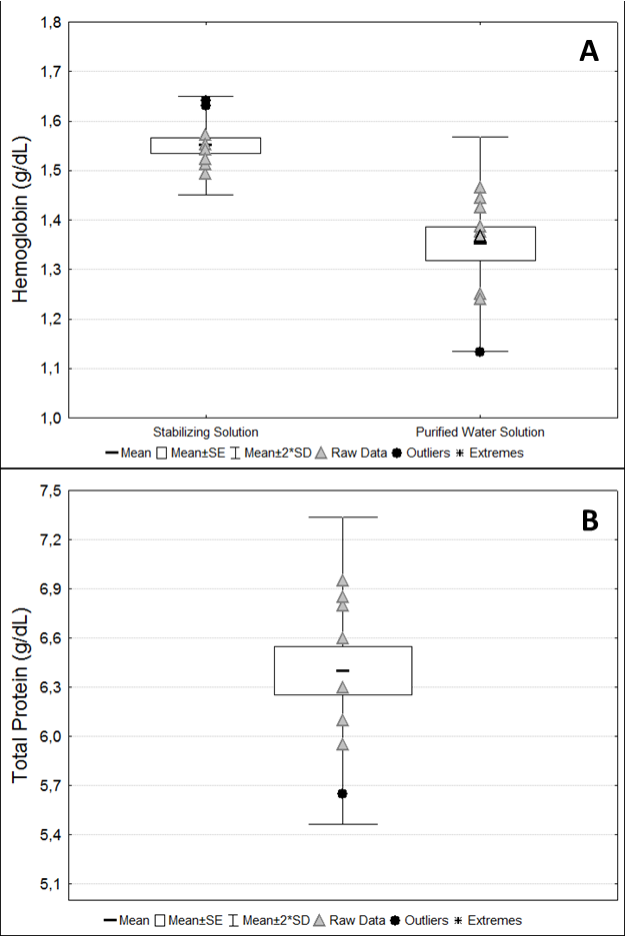
Protein analyses. (A) Hemoglobin concentration from samples diluted in stabilizing solution and purified water. (B) Total protein concentrations from serum samples. Raw data are presented for each marker.
TBARS and SH measurements were performed in washed erythrocytes samples diluted into purified water solution because the stabilizing solution interfered with the results of the assays ( Figure 10). The intra-assay coefficient of variation in all assays was considered low (up to 10%). CAT activity was not detected in the serum samples. Although sample volumes from 1 to 50 µL were tested for this assay, no activity was detected in healthy animals. Moreover, although concentrations around 0.044 mM of -SH per milligram of total protein in serum were detected, these concentrations were approximately 60-fold lower than those found in washed erythrocytes (2.74 mM/mg Hb) ( Figure 11).
  **Figure 10.**
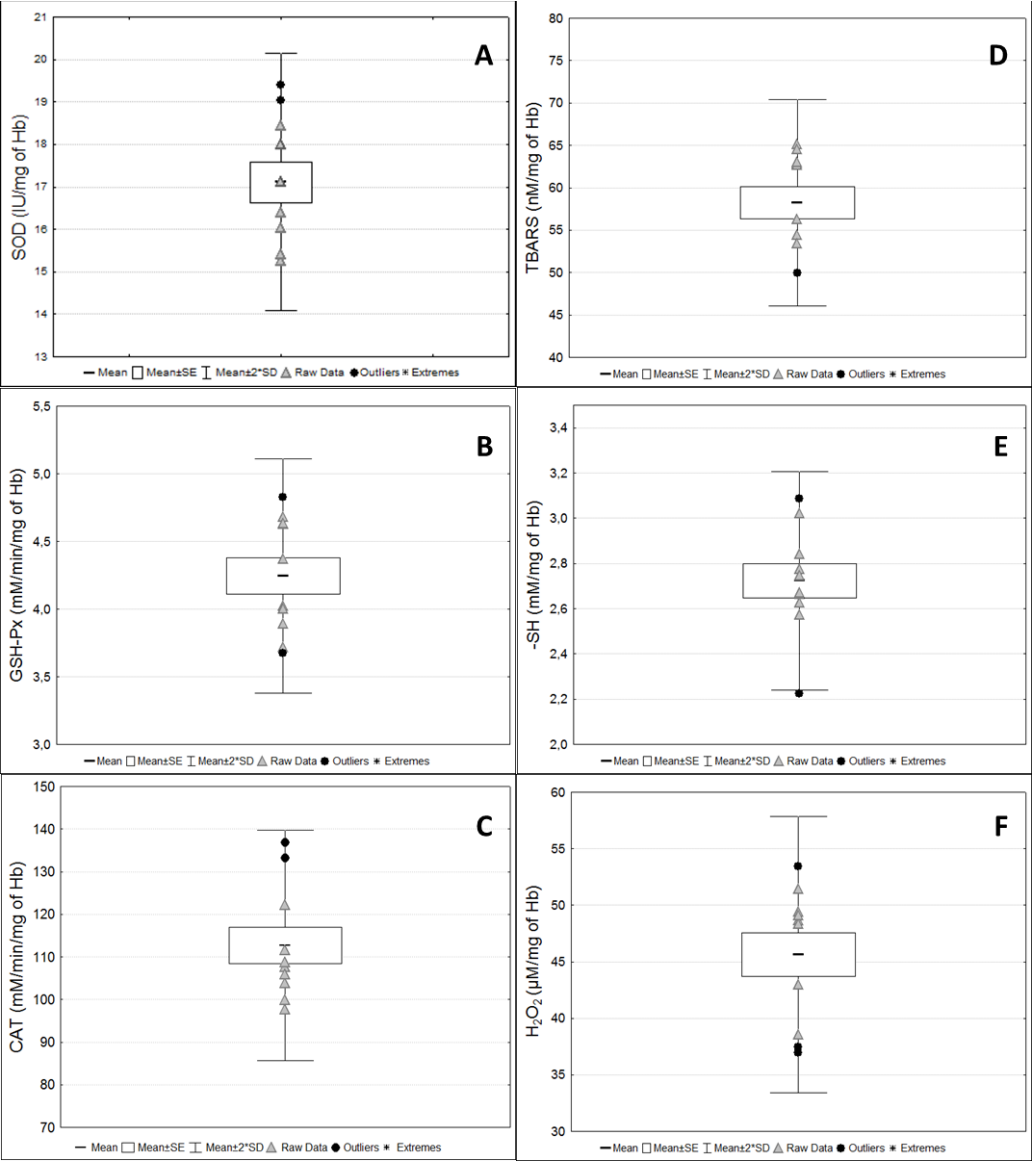
Biomarkers of oxidative stress status in the washed erythrocyte samples. Antioxidant enzyme activities and the redox status markers in washed erythrocyte samples from pregnant rats at term. (A) Superoxide dismutase (SOD); (B) Glutathione peroxidase (GSH-Px); (C) Catalase (CAT); (D) Thiobarbituric acid reactive substances (TBARS); (E) Reduced thiol groups (-SH); (F) Hydrogen peroxide (H _2_ O _2 _). Raw data are presented for each marker.
  **Figure 11.**
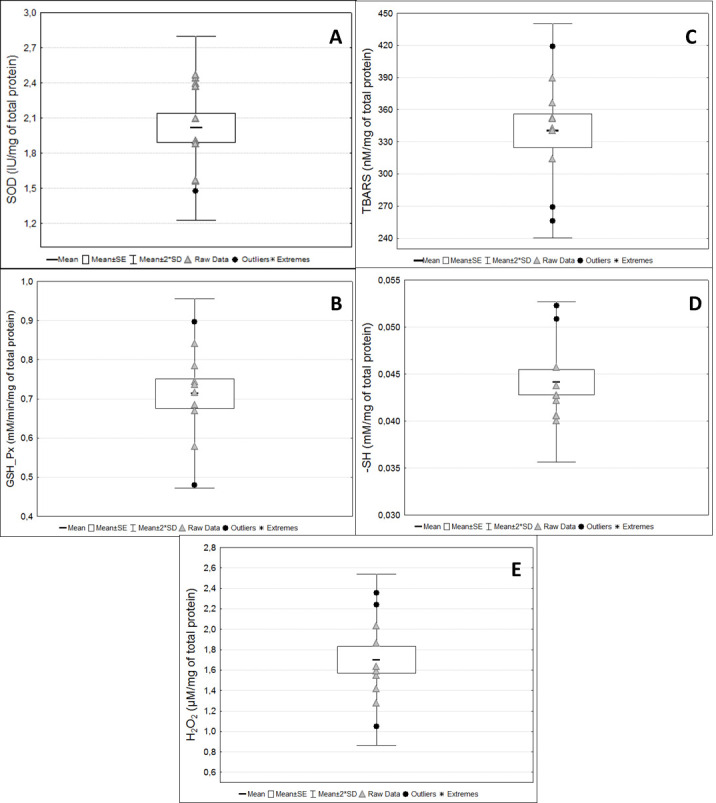
Biomarkers of oxidative stress status in serum samples. Activity of antioxidant enzymes and the redox status markers in serum samples from pregnant rats at term. (A) Superoxide dismutase (SOD); (B) Glutathione peroxidase (GSH-Px); (C) Thiobarbituric acid reactive substances (TBARS); (D) Reduced thiol groups (-SH); (E) Hydrogen peroxide (H _2_ O _2 _). Raw data are presented for each marker.

## Notes

1. Hemoglobin (Hb) concentration was determined and used to normalize the oxidative stress biomarkers (SOD, GSH-Px, CAT, -SH, TBARS, and H_2_O_2_) in washed erythrocytes according to the diluent used (purified water or stabilizing solution). Thus, hemoglobin concentration must be twice determined: in purified water and in stabilizing solution.
2. It is relevant to emphasize that the Hb and total protein concentrations should be transformed in mass (mg) in all formulas of oxidative stress biomarkers.
3. In TBARS assay, all the samples must be thawed at 25 °C. After TBA solution preparation, the pH should be below 2.0. If the pH is above 2.0, it is possible that the purified water conductivity is impaired.
4. In reduced thiol groups (-SH), hydrogen peroxide (H_2_O_2_), superoxide dismutase (SOD), GSH-Px, and CAT assays, all the samples must defrost on ice and be kept cooled to not allow degradation to occur.
5. Hydrogen peroxide (H_2_O_2_) is a volatile substance; thus, it is necessary to perform the equation for H_2_O_2_real molarity when executing the assay.
6. To reduce the sample volume and reagents to obtain the same results between microplates and cuvettes, the researchers decided to use these final equations of each biomarker following the light pathlength of cuvette methodologies (2 mL), but the sample and reagent volumes were adjusted to the microplate volume (well total volume 360 μL).

## Recipes

1. **
Stabilizing solution (2.7 mM EDTA and 0.7 mM 2-mercaptoethanol)
**
EDTA 10 mL
2-mercaptoethanol 0.5 μL
*
Note: Solution stable for up to six months at 2–8 °C.
*
2. **
Drabkin’s solution
**
Potassium ferricyanide 200 mg
Potassium cyanide 50 mg
NaHCO _3_ 1 g
Purified water 1,000 mL
*
Note: Drabkin’s solution is light sensible. Store at 4 °C in an Amber bottle to protect from light. This solution is stable for up to six months at 2–8°C.
*
3. **
1 μg/μL stock solution
**
Dilute 1 g of BSA in 1 mL of purified water.
*
Note: Solution stable for up to six months at 2–8 °C.
*
4. **
3% (w/v) 5-sulfosalicylic acid hydrate (for 100 samples)
**
Sulfosalicylic acid 3 g
Ultrapure water 100 mL
Dilute sulfosalicylic acid in a volumetric flask adding purified water.
Seal it with laboratory film.
Keep at 25 °C.
*
Note: Solution must be freshly prepared. After use, discard remaining solution.
*
5. **
Thiobarbituric acid solution (TBA) 0.67% (for 200 samples)
**
  - Dilute 0.67 g of TBA in 95 mL of purified water in an Amber bottle.
  - Keep the bottle partially sealed and heat the solution to 80 °C for 30 min in a water bath.
  - Wait until the solution reaches 25 °C.
  - Adjust pH to 2.0 by adding 1 M NaOH (see Recipe 16).
  - Add the solution to a beaker and complete to 100 mL using purified water.
*
Note: Solution must be freshly prepared at the assay day. This solution is light sensible. Store in Amber bottle. After use, discard remaining solution.
*
6. **
1 M HCl
**
Dilute 8.3 mL of HCl in 100 mL of purified water.
*
Note: Solution stable for undetermined time at 2–8 °C.
*
7. **
0.1 M TRIS/HCl 0.5 mM EDTA pH 8.0 (for 250 duplicate samples)
**
  - Dilute 1.2114 g of TRIS-Ultrapure ^TM^ and 0.0146 g of EDTA in 90 mL of purified water.
  - Adjust pH to 8.0 by adding 1 M HCl (see Recipe 6).
  - Transfer the solution to a beaker and complete to 100 mL using purified water.
*
Note: Solution stable for undetermined time at 2–8 °C.
*
8. **
10 mM DTNB (for 250 duplicate washed erythrocytes samples and 100 duplicate serum samples)
**
  - Dilute 0.0079 g of DTNB in 2 mL of ethyl alcohol.
  - Aliquot the total volume in two microtubes (1 mL each)
  - Seal and store at -20 °C.
*
Note: Solution must be freshly prepared and stored in a sealed tube at -20°C. After use, discard remaining solution.
*
9. **
Solution 1: 1.71 M NaCl, 34 mM KCl, 14 mM K_2_HPO_4_ , 0.1 M Na_2_HPO_4_**
  - Mix 8 g of NaCl, 0.2 g of KCl, 0.2 g of K_2_HPO_4_ , and 1.15 g of Na_2_HPO_4_ in a volumetric flask.
  - Add 80 mL of purified water and seal with laboratory film.
  - Keep at 25 °C until buffer solution B preparation (Recipe 15).
  - Store at 2–8 °C after buffer solution B preparation.
*
Note: Solution stable for undetermined time.
*
10. **
Solution 2: 90 mM CaCl_2_**
  - Dilute 0.3 g of CaCl_2_in 30 mL of purified water.
  - Homogenize and seal the solution with laboratory film.
  - Keep at 25 °C until buffer solution B preparation (Recipe 15).
  - Store at 2–8 °C after buffer solution B preparation.
*
Note: Solution stable for undetermined time.
*
11. **
Solution 3: 0.11 M MgCl_2_**
  - Dilute 0.3 g of MgCl_2_in 30 mL of purified water.
  - Homogenize and seal the solution with laboratory film.
  - Keep at 25 °C until buffer solution B preparation (Recipe 15).
  - Store at 2–8 °C after buffer solution B preparation.
*
Note: Solution stable for undetermined time.
*
12. **
Solution 4: 0.1% phenol red
**
  - Dilute 0.3 g of phenol red in 30 mL of purified water in a tube.
  - Homogenize the solution at 54 °C in water bath until complete dissolution.
  - Remove the solution from the water bath.
  - Seal the tube with laboratory film.
  - Keep at 25 °C until buffer solution B preparation (Recipe 15).
  - Store at 25 °C after buffer solution B preparation.
*
Note: Solution stable for undetermined time.
*
13. **
Phosphate buffer solution A
**
  - Add 2.61 g of K_2_HPO_4_ to 150 mL of purified water.
  - Homogenize and seal the solution with laboratory film.
  - Keep the solution at 25 °C.
  - Add 2.06 g of KH_2_PO_4_ to 150 mL of purified water.
  - Homogenize and seal the solution with laboratory film.
  - Keep the solution at 25 °C.
  - Add 100 mL of K_2_HPO_4_ solution in a volumetric flask and add KH_2_PO_4_ solution until reaching pH 7.0.
  - Store at 2–8 °C.
*
Note: Solution stable for undetermined time.
*
14. **
Peroxidase solution (HRP)
**
Dissolve 5 mg of HRP in 1 mL of phosphate buffer solution A.
*
Note: Solution must be freshly prepared. After use, discard remaining solution.
*
15. **
Buffer solution B
**
10.56 mL of purified water
0.960 mL of Solution 1
0.120 mL of Solution 2
0.120 mL of Solution 3
0.120 mL of Solution 4
0.120 mL of peroxidase solution (HRP)
*
Note: Solution must be freshly prepared. After use, discard remaining solution.
*
16. **
1 M NaOH
**
Dilute 0.040 g of NaOH in 0.10 mL of purified water.
*
Note: Solution stable for undetermined time at 2–8 °C.
*
17. **
1 M TRIS/HCl 5 mM EDTA pH 8.0 (for 150 duplicate samples)
**
  - Dilute 6.0570 g of TRIS-Ultrapure ^TM^ and 0.0731 g of EDTA in 45 mL of purified water.
  - Adjust pH to 8.0 by adding 1 M HCl (see Recipe 6).
  - Transfer the solution to a beaker and complete to 50 mL using purified water.
  - Store in a volumetric flask.
*
Note: Solution stable for undetermined time at 2–8 °C.
*
18. **
10 mM pyrogallol solution (for 250 duplicate sample analysis)
**
Dilute 0.0063 g of pyrogallic acid in 5 mL of 10 mM HCl solution (see Recipe 21).
*
Note: The solution must be freshly prepared. Minimum volume of 5 mL due to the pyrogallol quantity to be weighted. After use, discard remaining solution.
*
19. **
50 mM TRIS/HCl 5 mM EDTA pH 7.6 (for 500 duplicate sample analysis)
**
  - Dilute 0.6057 g of TRIS-Ultrapure ^TM^ and 0.1461 g of EDTA in 90 mL of purified water.
  - Adjust pH to 7.6 by adding 1 M HCl (see Recipe 6).
  - Transfer the solution to a beaker and complete the solution to 100 mL using purified water.
  - Store in a volumetric flask.
*
Note: Solution stable for undetermined time at 2–8 °C.
*
20. **
1% NaHCO _3_ and 1 mM EDTA
**
Dilute 1 g of NaHCO _3_ and 0.0292 g of EDTA to 100 mL of purified water in a volumetric flask.
*
Note: Solution stable for undetermined time at 2–8 °C.
*
21. **
10 mM HCl solution
**
Dilute 50 μL of HCl in 60 mL of purified water.
*
Note: Solution stable for undetermined time at 2–8 °C.
*
22. **
0.1 M glutathione reduced (GSH) (for 60 duplicate sample analysis)
**
Dilute 0.0184 g of GSH in 600 μL of 10 mM HCl solution (see Recipe 21).
*
Note: The solution must be freshly prepared and kept on ice. After use, discard remaining solution.
*
23. **
10 U/mL glutathione reductase (GSH-Rd) (for 60 duplicate sample analysis)
**
Add 60 μL of GSH-Rd to 2.940 mL of 0.1M TRIS/HCl 0.5 mM EDTA pH 8.0 (see Recipe 7).
*
Note: The solution must be freshly prepared and kept on ice. After use, discard remaining solution.
*
24. **
7 mM *t* -butyl hydroxiperoxide (for 125 duplicate sample analysis)
**
Dilute 5 μL of *t* -butyl hydroperoxide in 5 mL of purified water.
*
Note: The solution must be freshly prepared. After use, discard remaining solution.
*
25. **
4 mM NADPH (for 60 duplicate sample analysis)
**
Dilute 0.010 g of NADPH in 3 mL of 1% NaHCO _3_ 1 mM EDTA.
*
Note: Solution must be freshly prepared and kept on ice during the assay. Solution stable for up to 4 h.
*
26. **
0.1 M NaH_2_PO_4_ ·H_2_O (for 200 samples analysis)
**
  - Dilute 2.7598 g of NaH_2_PO_4_ ·H_2_O in 190 mL of purified water.
  - Adjust pH to 7.0 by adding 1 M NaOH (see Recipe 16).
  - Transfer the solution to a beaker and complete the solution to 200 mL using purified water.
  - Store in a volumetric flask.
*
Note: Solution stable for undetermined time at 2–8 °C.
*
27. **
1 M hydrogen peroxide (H_2_O_2_)
**
Add 970 μL of hydrogen peroxide to 9.03 mL of purified water.
*
Note: Solution must be freshly prepared. After use, discard remaining solution.
*
